# The contribution of electronic health records to risk management through accreditation of residential aged care homes in Australia

**DOI:** 10.1186/s12911-020-1070-y

**Published:** 2020-03-20

**Authors:** Ping Yu, Tao Jiang, David Hailey, Jun Ma, Siyu Qian

**Affiliations:** 1 Centre for IT-enabled Transformation, School of Computing and Information Technology, University of Wollongong, Wollongong, NSW 2522 Australia; 2 Illawarra Health and Medical Research Institute, Wollongong, NSW 2522 Australia; 3 SMART Infrastructure Facility, University of Wollongong, Wollongong, NSW 2522 Australia; 4 School of Nursing and Health Management, Shanghai University of Medicine and Health Sciences, Shanghai, 201318 China

**Keywords:** Accreditation, Electronic health records, Information system, Long term care, Nursing home, Nursing records, Risk management, Standard

## Abstract

**Background:**

The Australian government has implemented a compulsory aged care accreditation system to guide and monitor the risk management approach in registered residential aged care (RAC) homes. This research assessed the contribution of electronic health records (EHR) to risk management in RAC homes in relation to the extent that aged care accreditation fulfils its role.

**Methods:**

A convenience sample of 5560 aged care accreditation reports published from 2011 to 2018 was manually downloaded from the Accreditation Agency web site. A mixed-method approach of text data mining and manual content analysis was used to identify any significant differences in failure to meet accreditation outcomes among the RAC homes. This took account of whether EHR or paper records were used, year of accreditation, and size and location of the homes.

**Results:**

It appears that aged care accreditation was focused on structure and process, with limited attention to outcome. There was a big variation between homes in their use of measurement indicators to assess accreditation outcomes. No difference was found in outcomes between RAC homes using EHR and those using paper records. Only 3% of the RAC homes were found to have failed some accreditation outcomes. Failure in monitoring mechanism was the key factor for failing many accreditation outcomes. The top five failed outcomes were Human Resource Management, Clinical Care, Information Systems, Medication Management and Behavioural Management.

**Conclusions:**

Sub-optimal outcomes have limited the effectiveness of accreditation in driving and monitoring risk management for care recipient safety in RAC homes. Although EHR is an important structure and process component for RAC services, it made a limited contribution to risk management for accreditation in Australian RAC homes. Either EHR was not effective, or the accreditation process was not robust enough to recognize its influence. Aged care accreditation in Australia needs to develop further outcome-based measures that are supported by robust data infrastructure and clear guidance.

## Background

Residential aged care (RAC) services in Australia provide accommodation, meals, nursing and social care services for the frail older people by skilled and unskilled nursing staff [1, 2]. Associated with the ageing process are increased levels of frailty and chronic diseases, which are the main challenges for nursing staff to provide appropriate and safe care [2]. Generally, people living in RAC homes have higher exposure to various risk factors than their counterparts in the community [3]. These risk factors are related to residents’ personal health conditions, the health and aged care systems serving them and human factors from the medical and nursing staff [4].

### Risk management approach in residential aged care homes

In accordance with the international themes in health and personal services [5], a risk management approach has to be established in RAC homes to effectively control the risk factors for resident safety and wellbeing so as to comply with the legislative requirements in Australia. The process of risk management includes identifying and assessing risks, developing risk management plans, implementing risk management actions and re-evaluating emerging risks in the care processes [6].

An important objective of RAC homes is minimising risks to the individual care recipients, their families and society [7], which requires managing the risk factors related to residents’ health conditions. The classical nursing process model, which has been widely used as a theoretical framework for nursing practice and documentation in RAC homes in Australia, is a risk management approach to resident safety. This model is comprised of five stages: nursing assessment, nursing problem or diagnosis, planning, implementation and evaluation [8, 9]. Each stage of the model echoes the relevant stage in the risk management cycle. In the context of aged care nursing, ‘risk management’ encompasses many of the activities undertaken by qualified and non-qualified nursing staff [10]. An important objective of risk management in nursing practice is to reduce adverse events and incidents that under optimal conditions are not a normal consequence of a resident’s nursing and personal care.

To ensure the effective implementation of the risk management system to protect resident safety, Australian government has implemented a compulsory aged care accreditation program. This guides and monitors a risk management approach in registered RAC homes, similar to those in other OECD and European Union countries [11]. Aged care accreditation drives the risk management system in RAC homes in Australia, within six states and two territories: New South Wales (NSW), Victoria (VIC), Queensland (QLD), Western Australia (WA), South Australia (SA), Tasmania (TAS), Northern Territory (NT) and the Australian Capital Territory (ACT).

### Aged care accreditation

Aged care accreditation is a process that assesses quality of care and services provided in RAC homes, and gives recognition that providers are competent, comply with regulations and meet service quality standards [11]. It encourages quality and safety through a mix of compliance and quality elements, which can extend to continuous quality improvement. To ensure the effective function of the RAC risk management system and resident safety, the aged care accreditation system is implemented through the government-operated Australian Aged Care Quality Agency (AACQA), which determines whether the services provided by an RAC home meet the relevant safety standards. The AACQA commenced operation on January 1, 2014, superseding the Aged Care Standards and Accreditation Agency (ACSAA).

The AACQA makes use of the same four accreditation standards as its predecessor, both followed the Quality of Care Principles 2014 [12], made under the Aged Care Act 1997 [13]. An accreditation report is organised under four RAC accreditation standards: (1) management systems, staffing and organisational development; (2) health and personal care; (3) resident lifestyle; and (4) physical environment and safe systems [12]. The four standards include 9, 17, 10 and 8 expected outcomes, respectively, a total of 44 [12]. Common to all four standards are the outcomes of continuous improvement, regulatory compliance and education and staff development.

### The process of aged care accreditation

The process of aged care accreditation in Australia includes self-assessment by each RAC home against the accreditation standards and submission of an application for accreditation. This is followed by a desk audit and a site audit by a team of registered aged care quality surveyors. Then the AACQA publishes an official report, and makes the decision about whether the home met the standards. If the AACQA found that a service did not meet one or more outcomes under the relevant standards, it would require the service to rectify the non-compliance. Finally, an accreditation certificate is issued, when appropriate, and an accreditation report published on the AACQA’s web site [14].

### Information systems in RAC homes

Information management is fundamental to healthcare delivery. A variety of information systems, covering client registration, billing and client health records, have been established in Australian RAC homes to support a variety of organisational purposes and functions. These include administrative, financial, regulatory compliance, care delivery and quality assurance. As the communication tool for information exchange between nursing staff and with outside health service providers [15], the client health record system, either in paper or electronic format, is the most important information system in RAC homes. It supports the delivery of aged care services and risk management for client safety, promotes effective communication between caregivers, and facilitates continuity and individuality of care of clients, and regulatory compliance [8, 9].

By the end of 2014, 37.4% (1031 of 2756) of the accredited RAC homes in Australia had introduced electronic health records (EHR) to manage client health and lifestyle information and to improve effectiveness and efficiency in information management [4]. Although EHR is a major technology to support the delivery of aged care services and risk management for client safety in RAC homes, its rate of adoption appeared to have stagnated since 2014 according to our analysis of the publicly - available aged care accreditation reports.

The study described here aims to research the effectiveness of EHR in risk management for accreditation in RAC services in Australia. In order to provide contextualised and valid explanations of the findings, the following research questions were addressed.

- What were the measurement indicators used by the accreditation agency to assess the accreditation outcomes? Were the indicators consistently implemented?
- What were the risk indicators for RAC homes to fail the accreditation outcomes?
- Which accreditation outcomes were RAC homes mostly likely to fail?
- Were there any differences in accreditation failure rate after the accreditation authority changed from ACSAA to AACQA?
- Were there any differences in accreditation failure rate between the RAC homes using EHR and those with paper records, over accreditation years, facility size, location, or ownership?

## Methods

A secondary research was conducted on the publicly available aged care accreditation reports published by Australian Aged Care Quality Agency (AACQA) from 2011 to 2018. As the study does not include human subjects, it does not require ethics approval.

Text data mining was conducted following a four-step process we developed in a previous study [4]: data sourcing and processing, development, test and use of a computer program for data extraction, data labelling and data analysis.

### Step 1. Data sourcing and processing

A convenience sample of 5560 aged care accreditation reports published from 2011 to 2018 was sourced from the AACQA website (www.aacqa.gov.au). The first collection was conducted in 2015. All 3607 reports published between March 7, 2011 and March 7, 2015 were downloaded. The second collection was conducted in 2019. A stratified sample of reports were downloaded for each state or territory. The simple random sampling method, i.e., randomly picking a number, was applied to select half of the reports that were published between March 8, 2015 and December 31, 2018. After removing those reports that could not be opened or did not contain actual findings, 1953 reports were collected. Each report was about 20–30 pages.

These reports were published in Microsoft word or pdf formats. The word documents were converted to pdf files, which were further converted to text files using custom-built software operating in conjunction with Adobe Acrobat XI Version 11.0.23. Errors generated during conversion, i.e., mis-spelling, broken line and unnecessary symbols, were fixed manually in comparison with the original pdf documents. The incorrect character encoding was concentrated on list characters like ‘•’ in pdf format. As all errors did not influence reading the content, they were converted to ‘?’ or ‘????’ in the text files.

### Step 2. Development, test and use of a computer program to extract data

A computer program was developed to automatically extract data using keywords. For example, to extract data on whether an RAC home used EHR, the keywords used included ‘electronic clinical plan’, ‘electronic clinical documentation’, ‘electronic clinical information’ and ‘electronic care plan’. To identify whether the home failed an expected outcome, the keywords ‘not met’ and the title of that outcome were used.

The accuracy of the computerised conversion function was tested by manual comparison with human judgement for a set of 100 reports randomly selected from the first batch of 3607 reports. As no difference in results was found between the two methods, the program was effective to extract the data, thus was applied to all the reports.

### Step 3. Data labelling

Data items extracted from each report included (1) the jurisdiction where the RAC home was located. (2) reporting year; (3) size of the RAC home (large, ≥ 60 beds or small < 60 beds); (4) type of information system used by the home (EHR or paper); (5) accreditation outcome of the RAC home (failed or passed); and (6) accreditation result of each of the 44 expected outcomes (labelled by 0 [not met] or 1 [met]). Ownership, whether not-for-profit, private or government owned, of the RAC homes that failed the accreditation was identified by manually searching online.

The extracted data were stored in a Microsoft Excel spreadsheet. Each row stored the data extracted from one accreditation report. Therefore, there were 5560 rows plus one header row in this spreadsheet.

### Step 4. Data analysis

#### Quantitative analysis

The unit of analysis was an accreditation report. The data were imported into SPSS version 21.0. Pearson’s chi-square test was conducted to identify any significant differences in the percent of RAC homes failing accreditation outcomes between electronic or paper records, years, home size and location. Descriptive analysis was also conducted. The difference was significant if *p* < 0.05.

#### Qualitative analysis

Qualitative analysis was conducted to understand which indicator was used to assess if an RAC home did or did not meet a specific accreditation outcome, and how the indicator was applied across the country.

##### Evaluating the indicators used by the accreditation agency to assess the accreditation outcomes

Of the 5560 reports, 5394 announced an RAC home had met all the accreditation outcomes. In order to identify the measurement indicators used by the Agency to assess the accreditation outcomes, about 9% (511) of these 5394 reports were stratified for qualitative analyses. The stratification followed the principle of a balanced ratio of reports from different years, facility size (small or large), using EHR or paper, and location in different jurisdictions. In each stratum, the simple random sampling method was applied to select the reports.

The reporting statements for two accreditation outcomes, 1.8 Information Systems and 2.4 Clinical Care, were analysed in the following steps.

Step 1. Developing coding statement applying an inductive, qualitative constant comparison method. First, we reviewed all the accreditation statements in one report to identify semantically similar and different ones, and coded them in an Excel table. Then we compared the statements in the second report with the codes. If the meaning was different, a new code was added. This process continued with the subsequent 50 reports. As no further code could be identified, the coding was saturated and stopped.

Step 2. Mapping the accreditation statements to the standard codes. The written statements in each accreditation report were mapped to the coding statements developed in Step 1. If the meaning was same or was synonymous with a coding statement, then Boolean value ‘1’ was recorded to represent presence, otherwise ‘0’ was recorded.

Step 3. Performing Pearson’s chi-square test to detect any difference in the ratio of presence of a statement in the accreditation reports between the RAC homes using EHR and paper, large and small, and located in different states. Due to smaller number of reports from the ACT, NT, TAS and WA, data from these jurisdictions were combined to enable meaningful statistical analysis.

##### Identification of the accreditation outcomes that the RAC homes most likely to fail

Only 166 of all 5560 reports (3%) announced an RAC home failing one or more accreditation outcomes. 45 were ACSAA reports, 121 were AACQA reports. All 45 ACSAA reports and 12 randomly selected AACQA reports were analysed. Overall, 34% (57/166) of the reports were analysed.

## Results

### The measurement indicators for accreditation outcome 1.8 information systems

In total, fifteen indicators were used to assess the accreditation outcome 1.8 Information Systems. Further details of the findings for these indicators are provided in Additional file 1.

#### Comparison between the ACSAA and AACQA reports

The AACQA reports give an indication of Australian aged care accreditation practice in recent years. There was an increased presence of five indicators covering staff access to information, satisfaction with information management, whether information is up-to-date, accuracy of information, and education of staff. There was a decrease from levels in the ACSAA reports for presence of three indicators on information communication to staff, information communication to residents and information archiving and disposal.

Two indicators were consistently implemented in the majority of the AACQA accreditation reports. The first was ‘Information collection, management and storage are conducted in a secure and confidential manner’, in 96.9% of the AACQA reports. The other was ‘Staff have access to information (e.g. care plans, policies) appropriate to their role with appropriate access control’, presented in 80.9% of the reports. Another five indicators were reported in more than 50% of the AACQA reports (Additional file 1).

The presence rates for eight of the 15 indicators were less than 50%, reflecting a high variation in their implementation in Australian RAC homes. Only 35.1% of the AACQA reports mentioned ‘Information is accurate’, despite this being the vital measure of information quality. The lowest reporting rate of 8% was found for ‘Issue reporting process is in place for continuous improvement, regulatory compliance and other relevant aspects of service’, though this was double the rate shown in the earlier ACSAA reports.

#### Use of information systems, size and location of RAC homes

No difference was found in the presence of indicators to assess the RAC homes with different types of information systems (EHR or paper). The indicator of information up-to-date was reported more often in the large RAC homes than in the small ones (38.4% vs 28.7%). There were also significant differences between jurisdictions. For example, there was a significant difference between VIC and NSW reports in mention of 4 of the 15 indicators.

### The measurement indicators for accreditation outcome 2.4 clinical care

#### Comparison between the ACSAA and the AACQA reports

There was a significant difference between the ACSAA and AACQA reports in the inclusion of 10 of the 17 accreditation indicators for outcome 2.4 Clinical Care (Additional file 1). There was increased reporting in the later reports for eight indicators, and a significant reduction in the reporting of two others. The two most frequently reported indicators in the AACQA reports, were ‘Residents and representatives are satisfied with the clinical care provided’ (83.2%); and ‘Management monitors clinical care through audits, clinical data analysis, monthly care plan reviews and consultation with the resident and/or their representative’ (79.0%).

Overall, seven criteria measuring 2.4 Clinical Care appeared in 49 to 83% of the AACQA accreditation reports. The other eight criteria had a high variation, from 6 to 47%, in their appearance. The least presented criterion was ‘The home has appropriate supplies of equipment and resources that are maintained in good working order to meet the ongoing needs of care recipients’; appeared only in 6% of the reports.

#### Use of information systems, size and location of RAC homes

No difference was found between RAC homes with EHR and those using paper in the accreditation indicators justifying acceptance that a home met outcome 2.4 Clinical Care. Nor was there a difference between large and small RAC homes in the acceptance of that outcome (Additional file 1). However, some significant differences were found between jurisdictions. For example, the QLD reports mentioned the indicator ‘Residents’ care needs are assessed on entry to home’ more often than the NSW reports (76.6% vs 52.5%).

### Risk indicators for RAC homes failing the accreditation outcomes 1.8 and 2.4

Ten indicators were used in the accreditation reports to determine that an RAC home did not meet the accreditation outcome 1.8 and 14 for outcome 2.4 (Table 1). Failure in monitoring mechanisms was a common reason for RAC homes to fail seven accreditation outcomes (Table 2). There were four major reasons for RAC homes failing medication management accreditation outcomes (Table 3).

**Table 1:** Risk indicators used to determine RAC homes failing accreditation outcomes 1.8 and 2.4

**Indicators used to determine that outcome 1.8 Information Systems was not met**	**Indicators used to determine that outcome 2.4 Clinical Care was not met**
1. Information was not accurate, up-to-date, complete, consistent and sufficient to guide care delivery. 2. Staff did not have access to the information. 3. Monitoring mechanisms were not effective in identifying deficiencies in management and staff practice. 4. Staff did not consistently report clinical incidents. 5. Key information such as clinical data was not always used to identify and meet the needs of residents and staff. 6. Communication was not systematic and effective. 7. Policies and procedures were not evidence-based. 8. Staff were not familiar with computerised systems. 9. Management did not regularly review the information system. 0. Residents and their representatives were not satisfied because the information they provided were not incorporated into care.	1. RAC home was not able to demonstrate residents were receiving appropriate clinical care. 2. Staff did not appropriately assess residents’ clinical care needs on entry to home and on an ongoing basis. 3. Care plan did not always reflect current care needs and preferences of residents. 4. Clinical care was not provided in accordance with directives from qualified staff, care plan and residents’ needs and/or preferences. 5. Changes in care were not consistently documented and doctors were not informed about them. 6. Residents experienced unrelieved pain, their wounds were not always promptly reviewed, and changes in their health conditions were not recognised by staff. 7. Information was not always current, accurate and consistent. 8. Information systems and communication processes were not effective to support the provision of appropriate clinical care. 9. Monitoring mechanisms such as audits, care reviews and incident reporting were not effective in the identification of deficiencies in care delivery. 10. Clinical incidents were not consistently collated and analysed to identify opportunities for improvement. 11. Referrals to other health professionals were not implemented in a timely manner. 12. Staff was not knowledgeable about residents’ care needs. 13. Staff reported not adequate time to complete all required tasks and staffing insufficiency had negative impact on their ability to provide care that meets care recipients’ needs and preferences. 14. Residents and representatives reported dissatisfaction with the adequacy and responsiveness of care.

**Table 2:** Failed accreditation outcomes where monitoring mechanism was cited as a cause of failure

**Failed outcome**	**Statement that cited monitoring mechanism failure**
1.6 Human Resource Management	Did not have effective monitoring systems in place to identify deficits in staff skills and practices.
2.7 Medication Management	Failure to ensure safe and secure medication management
2.8 Pain Management.	Monitoring mechanisms not effective in ensuring residents’ pain management needs
2.13 Behavioural Management	Problems in the identification and reporting of episodes of challenging behaviours
3.6 Privacy and Dignity	Lacked an effective system to monitor the maintenance of this area.
2.10 Nutrition and Hydration	Lacked consistent monitoring of residents’ weights as required by the care plan.
2.5 Specialised Nursing	Registered nurses did not monitor residents’ specialised nursing care needs, causing a failure in information systems to identify the change in a resident’s health status.

**Table 3:** Major indicators for RAC homes failing medication management outcome

**No.**	**Indicators**
1	Residents’ medications were not managed safely and correctly.
2	Medications were not consistently administered in accordance with medical officer prescription.
3	Processes were not effective in ensuring maintenance of sufficient stock of regular medications and timely commencement of short term medications.
4	Monitoring processes did not identify incorrectly stored medications.

### Comparison of the ACSAA and AACQA reports

Comparison of details for the two series of reports is shown in Table 4. The failure rate for RAC homes was higher in the AACQA than the ACSAA reports, while mean numbers of outcomes failed were similar. There were significantly higher failures rates in the AACQA series for 28 of the 44 accreditation outcomes considered, and no significant difference from the ACSAA reports for the remainder. Four of the five most frequently failed outcomes were common to both series of reports. The number of reports recorded failure of each of the 44 accreditation outcomes is presented in Additional file 1.

**Table 4:** Comparison of findings in ASCAA and AACQA reports

	**ASCAA**	**AACQA**
Number of reports	2684	2876
Failure rate	1.2%	4.6%
Mean number of outcomes failed	5.5	5.6
Most frequently failed outcomes	Information Systems (0.7%)	Human Resource Management (2.2%)
	Clinical Care (0.6%)	Clinical Care (1.9%)
	Medication Management (0.5%)	Information Systems (1.8%)
	Human Resource Management (0.4%)	Medication Management (1.5%)
	Continuous Improvement (0.4%)	Behavioural Management (1.1%)

### Failure rate and use of information systems

There was no significant difference in the failure rate between the RAC homes using an EHR system (2.8%, 55/1988) and those using a paper-based system (3.1%, 111/3572). The failure rate varied over the years, increasing from 2014 to a highest value of 12.1% in 2018. As indicated in Table 5, there did not appear to be a clear relationship between failure rate and the proportion of reports for homes that used EHR. Increased use of EHR did not seem to bring a clear risk management benefit through decreasing accreditation failure rates.

**Table 5:** Accreditation failure rate and use of EHR

**Year**	**2011**	**2012**	**2013**	**2014**	**2015**	**2016**	**2017**	**2018**
Failure rate, %	2.9	0.8	1.2	0.6	2.1	4.0	7.2	12.3
Proportion of reports from homes with EHR, %	29.5	31	32.9	36	43.3	52.4	42.1	31.1

### Size of RAC homes

For those RAC homes that used EHR, large homes had a significantly higher failure rate than small ones. For large homes, the failure rate was lower for those with EHR than those using paper. For small homes the difference between failure rates for EHR and paper was less (Table 6).

**Table 6:** Failure rates by home size and type of record

**Type of home and records**	**Number of reports**	**Number of failures**	**Failure rate**
Large home, EHR	1169	40	3.4%
Large home, paper	2025	80	4.0%
Small home, EHR	819	15	1.9%
Small home, paper	1547	31	2.0%

### Location of RAC homes

There was some variation between jurisdictions in the failure rates for their RAC homes, with QLD having a higher rate and VIC a lower rate than the others (Table 7).

**Table 7:** Failure rates in different jurisdictions

**Jurisdiction**	**NSW**	**VIC**	**QLD**	**SA**	**Others**^**a**^
Number of reports	1668	1631	986	512	763
Failure rate, %	3.2	1.8	4.5	3.1	3.0

^a^WA, TAS, ACT, NT

### The ownership of the RAC homes that failed the accreditation outcomes

The 166 reports that recorded failure were from 155 RAC homes. Of these, 61.9% were not-for-profit, 29.7% were private and 8.4% were government - owned. 93.5% of these RAC homes failed accreditation outcome once, 5.8% failed twice and one failed three times (Additional file 1).

## Discussion

An important objective of RAC services is minimising risks to the individual care recipients, their families and society [7]. This objective is a key driver for the introduction of EHR in RAC homes [4]. To protect care recipient safety, the Australian government has implemented mandatory aged care accreditation in government registered and subsidised RAC homes. Aged care accreditation drives and monitors the risk management practice in RAC homes in Australia. It produces systematic, standardised national reports about the status of RAC homes in meeting the minimum aged care service standards. These reports provided solid data for addressing the aim of this study, evaluating the contribution of EHR to risk management through accreditation in RAC homes in Australia.

Using a mixed method approach of text data mining and manual text data analysis, 5560 aged care accreditation reports published between March 7, 2011 and December 31, 2018 were analysed. The conduct of Australian aged care accreditation was evaluated through itemized comparison of the measurement indicators for two accreditation outcomes 1.8 Information Systems and 2.4 Clinical Care. The risk indicators for RAC homes to fail these accreditation outcomes were also considered. These provide a suitable context for evaluating the contribution of EHR to risk management in RAC. It takes into consideration the difference between two types of accreditation reports (ACSAA and AACQA).

### Aged care accreditation in Australia fell short in assessing safety

Across the OECD countries, external regulatory control over RAC services is typically focused on controlling input (physical and human resources) by setting minimum acceptable standards and enforcing compliance [11]. Typically, accreditation requirements involve benchmarks for structure, workforce and safety. These aspects are reflected in Australian aged care accreditation by the structural component Information Systems and the workforce component Human Resource Management. These had always been among the top five failed accreditation outcomes.

However, aged care accreditation in Australia appears to fall short in assessing safety. There was only one safety-related indicator for the accreditation outcome 1.8 Information System: “Residents and representatives are satisfied with the information management of the home”, and one for 2.4 Clinical Care: “Residents and representatives are satisfied with the clinical care provided”. These two outcome measurement statements are subjective, vague and difficult to quantify. This echoes the observation of Nies et al. [11] that enforcement of external accreditation regulatory controls might be lenient in the OECD countries, therefore quality assurance needs to transfer to outcome-based measures that are supported by robust data infrastructure and clear guidance.

### Use of measurement indicators to assess accreditation outcomes in ACSAA and AACQA reports

There was an improvement in the consistency of use of measurement indicators for quality assessment after the transition of the accreditation authority from ACSAA to AACQA in 2014. This was indicated by a significantly increased use of 33.3% of the measurement indicators to assess accreditation outcome 1.8 Information Systems and 47.1% of the indicators for outcome 2.4 Clinical Care. However, there remained a huge variation in the implementation of more than half of the indicators. This suggested a big variation in the implementation of quality standards by accreditation surveyors across the country. This might reflect a lack of robust data infrastructure and clear guidance to the quality surveyors on which indicator data to capture and how to accurately and fully use clear indicators to assess the quality of RAC services.

### Risk indicators for RAC homes to fail accreditation outcomes 1.8 information systems and 2.4 clinical care

Failure in monitoring mechanism is the key factor for RAC homes to fail many accreditation outcomes. In our previous study, six risk indicators to fail the information system accreditation outcome were summarized for the RAC homes that used paper records [4]. In this study, nine risk indicators were extracted, including five of those identified in the previous study. As many more RAC homes have failed the accreditation outcome 1.8 Information Systems since 2015, it is reasonable to expect this increased identification of risk indicators for information system failure.

There were fourteen risk indicators for RAC homes to fail the Clinical Care accreditation outcome. Five revealed deficiency in nursing process. The close proximity of the nursing process model to the risk management cycle supported a failure of risk management for clinical care. Two risk indicators suggested information system failure: information was not always current, accurate and consistent; and information systems and communication processes were not effective to support the provision of appropriate clinical care. This echoes the importance of the information system for clinical care.

### The accreditation failure rate

Of the 5560 aged care accreditation reports published in 8 years, only 3% (166) reported failure of one or more accreditation outcome. Failure rate was the lowest in 2014 when AACQA took over from ACSAA. Over the following 4 years the failure rate then increased to reach 12.3% in 2018. There were also more failed accreditation outcomes in the AACQA reports than in the ACSAA reports.

What is concerning is a continued increase in the failure rates in the core safety areas of Clinical Care, Medication Management and Behavioural Management over the years. Large RAC homes had a significantly higher failure rate than the small ones. Failure rates varied between jurisdictions The RAC homes in QLD had a higher failure rate, and those in VIC a lower rate, than homes in other jurisdictions. Possible explanations for these variations are that the implementation of measurement indicators was different across the years and locations, or that the quality of RAC services was indeed different.

### The extent of aged care accreditation in fulfilling its role of guiding and monitoring risk management practices

Meeting accreditation standards is a mandatory requirement for the RAC homes in Australia to acquire registration and to receive government subsidy for care services. Accreditation drives the risk management approach in RAC homes to providing safe care for their residents. Our findings suggest that the effect of accreditation on driving and monitoring the risk management approach in RAC services was sub-optimal.

A risk-based approach to accreditation needs to take proper account of relevant history and current risk profile of a facility, to seek the true views of care recipients and their representatives, and to ensure that quality surveyors have adequate competency for the job [16]. In the period covered by this study, the Australian aged care regulatory system has been fragmented [16]. Responsibility was split between the Department of Health, the Quality Agency and the Complaints Commissioner, creating opportunity for miscommunication or discoordination between the three organisations. There was a lack of mechanism for Aged Care Accreditation Agency to identify ‘at risk’ RAC services.

In this study, the unit of analysis was an individual accreditation report. There were cases where several reports were produced for one RAC home. 5.8% of the 155 RAC homes that failed the accreditation outcomes were sampled twice and one was sampled three times. As the rate of repetitive failure in accreditation for an RAC home was minimal, it did not appear that the accreditation took adequate account of the relevant history and risk profile of an RAC home in the previous accreditation audit.

Another factor that may reduce the rigor of accreditation was that some RAC services became adept at providing the quality surveyors with “the look and feel” and information that the surveyors would expect, instead of presenting a true profile that the care recipients experienced on daily basis [16].

### Lack of evidence of contribution of EHR to risk management for accreditation in RAC homes

Only a few OECD countries have well-established information systems for care quality in RAC homes. Australia appears to be advanced in that respect, with 31 to 52% of the sampled RAC homes using EHR in the last 5 years. However, there was a similar level of accreditation failure rates in RAC homes using EHR and those using paper records. This might be due to a failure of accreditation in assessing outcomes, particularly a lack of coverage of safety outcomes. Accreditation may not be robust enough to identify the performance difference brought about by the use of EHR to replace paper records. Another possibility is that both EHR and paper records were weak in documenting measurable and concrete resident outcomes and nursing processes [17], and thus of limited quality [18–20].

Observational studies in Australian RAC homes have found that oral communication was the most frequent activity and the major communication channel between nursing staff, with residents and with outside service providers [2, 21–23]. About two-thirds of activities of nursing staff had a duration of 1 minute or less [2]; therefore nursing staff rarely had time to consult nursing records in the care delivery process [1]. Even in conducting high risk activity of medication management that requires clear information, the nursing staff only spent one to 2 minutes on reading residents’ records in a three-hour round [24]. EHR systems may not be adequately used in RAC homes to support nursing staff delivering care services, nor risk management to ensure resident safety.

## Limitations

This was a secondary study, with all the data drawn from the accreditation reports that were made publicly available on the AACQA’s web site. Due to resource limitation, comprehensive, detailed qualitative analysis of the measurement indicators was focused on 1.8 Information Systems and 2.4 Clinical Care, two accreditation outcomes with the highest failure rates in the ACSAA reports.

We could only investigate whether an RAC home had met a specific accreditation outcome. It was not possible to compare the quality of services among RAC homes once they met the minimum accreditation standards as the accreditation reports did not provide information on practice details of the sort that could be obtained from observational studies. We can decide whether an RAC used an EHR system or paper for information management but cannot identify the specific type of EHR system used in an RAC home as it was not described in the accreditation reports. We extracted the major indicators for failing other accreditation outcomes, but did not analyse all the measurement statements for all 44 accreditation outcomes.

Our findings demonstrate an increasing percentage of RAC homes failing aged care accreditation outcomes since 2015. However, we cannot ascertain the cause for the increase. Possibilities are a more stringent implementation of quality measurement standards or a genuine quality deterioration in the RAC services.

As only 166 reports documented failure in one or more expected outcomes, the evidence collected from this information source is still limited. Nevertheless the strength of the study is that it provides a nationwide overview of why Australian RAC homes failed accreditation outcomes in an 8 year period. It gives some insights into aspects of risk management and safety-related issues in RAC homes which may be helpful for improvement of aged care accreditation structure and processes.

## Conclusion

No difference in accreditation outcomes was found between the RAC homes using EHR or paper records. Accreditation was mainly focused on structures and processes, rather than on outcomes. There was also a big variation on the use of measurement indicators to assess accreditation outcomes. It remains uncertain whether the findings indeed reflected a lack of effect of EHR on risk management for aged care accreditation, or were due to a failure of accreditation in detecting the positive effect of EHR in supporting aged care services.

### Implication for practice

In order to effectively fulfil the role of guiding and monitoring risk management practices in RAC homes, aged care accreditation in Australia needs to transform to outcome-based measures that are supported by robust data infrastructure and clear guidance. The EHR system and its use may also need to be improved to truly support risk management for accreditation in RAC homes.

## Supplementary information

**Additional file 1.**

## Data Availability

The datasets generated and/or analysed during the current study are available on the Australian Aged Care Quality Agency website: www.aacqa.gov.au The source code for the processing systems and the extracted data set are available from the authors on request.

## Abbreviations

RAC: Residential aged care
ACSAA: Aged Care Standards and Accreditation Agency
AACQA: Australian Aged Care Quality Agency
EHR: Electronic health records
NSW: New South Wales
VIC: Victoria
QLD: Queensland
WA: Western Australia
SA: South Australia
TAS: Tasmania
NT: Northern Territory
ACT: Australian Capital Territory
