# Supplementary material for: The contribution of electronic health records to risk management through accreditation of residential aged care homes in Australia
Source: BMC Med Inform Decis Mak. 2020 Mar 20;20:58. doi: 10.1186/s12911-020-1070-y (PMC7082951; doi:10.1186/s12911-020-1070-y)
Supplement: Supplementary file 1 — Additional file 1. [file 12911_2020_1070_MOESM1_ESM.docx]

# Appendix 1. A comparison of the percentage of presence of the measurement indicators for the accreditation outcome 1.8 Information Systems

| **Accreditation indicator** | **Type of reports** | | **Type of information system** | | **Size of RAC homes** | | **State** | | | | |
| --- | --- | --- | --- | --- | --- | --- | --- | --- | --- | --- | --- |
|  | **ACSAA** | **AACQA** | **EHR** | **Paper** | **Small** | **Large** | **NSW** | **VIC** | **QLD** | **SA** | **ACT, NT, TAS, WA** |
| **Number of reports analysed** | **249** | **262** | **161** | **350** | **209** | **302** | **158** | **137** | **77** | **53** | **86** |
| 1. Information collection, management and storage are conducted in a secure and confidential manner | 95.6% | 96.9% | 95.0% | 96.9% | 94.7% | 97.4% | 94.9% | 97.8% | 100% | 96.2% | 93% |
| 2. Staff have access to information (e.g. care plans, policies) appropriate to their role with appropriate access control | 52.6% ^a^ | 80.9% ^b^ | 70.8% | 65.4% | 67.0% | 67.2% | 53.2%^b^ | 76.6%^a^ | 71.4%^a,b^ | 69.8%^a,b^ | 72.1%^a^ |
| 3. Information is communicated to staff via a variety of means such as memoranda, notice boards, handover processes and residents' clinical files | 84.3% ^a^ | 77.5% ^b^ | 80.7% | 80.9% | 82.8% | 79.5% | 83.5% | 82.5% | 72.7% | 73.6% | 84.9% |
| 4. Residents and representatives are satisfied with the information management of the home | 40.6% ^a^ | 71.0% ^b^ | 55.9% | 56.3% | 54.5% | 57.3% | 38.6%^b^ | 58.4%^a^ | 83.1%^c^ | 71.7%^a,c^ | 51.2%^a, b^ |
| 5. Information archiving and disposal follow appropriate policies | 83.9% ^a^ | 67.6% ^b^ | 80.1% | 73.4% | 76.6% | 74.8% | 75.3% | 74.5% | 71.4% | 83.0% | 76.7% |
| 6. Information is communicated to residents and representatives via a variety of means such as newsletters, resident meetings, resident advocate and feedback sheet | 95.2% ^a^ | 50.0% ^b^ | 69.6% | 73.1% | 74.6% | 70.2% | 77.8%^b^ | 70.8%^a,b^ | 58.4%^a^ | 77.4%^a,b^ | 72.1%^a,b^ |
| 7. Information is up-to-date | 17.7% ^a^ | 50.4% ^b^ | 32.9% | 35.1% | 28.7% ^a^ | 38.4% ^b^ | 26.6%^b^ | 47.4%^c^ | 45.5%^a,c^ | 22.6%^a,b^ | 25.6%^a,b^ |
| 8. Information is reviewed regularly to ensure currency and appropriateness | 35.7% | 42.0% | 35.4% | 40.6% | 41.1% | 37.4% | 38.6%^a,b^ | 43.8%^a,b^ | 24.7%^b^ | 49.1%^a^ | 38.4%^a,b^ |
| 9. Information is backed up regularly to prevent loss of information | 36.1% | 39.3% | 37.3% | 38.0% | 38.8% | 37.1% | 38.0% | 40.1% | 31.2% | 39.6% | 38.4% |
| 10. Information is accurate | 16.9% ^a^ | 35.1% ^b^ | 26.7% | 26% | 25.4% | 26.8% | 17.7%^b^ | 32.8%^a^ | 39%^a^ | 26.4%^a,b^ | 19.8%^a,b^ |
| 11. Staff are educated with information confidentiality and sign on confidential agreement | 14.5% ^a^ | 34.0% ^b^ | 24.8% | 24.3% | 24.4% | 24.5% | 25.9%^a,b^ | 19%^b,c^ | 28.6%^a,b^ | 3.8%^c^ | 39.5%^a^ |
| 12. Information system is monitored through feedback, audit, comments and complaints | 23.7% | 19.5% | 22.4% | 21.1% | 21.5% | 21.5% | 13.9%^a,b^ | 11.7%^a^ | 28.6%^b,c^ | 52.8%^c^ | 25.6%^a,b^ |
| 13. Management routinely collate, analyse, review and table relevant information from clinical records and indicators, monitoring and reporting mechanisms and human resource processes for continuous improvement | 15.7% | 12.2% | 13.0% | 14.3% | 15.8% | 12.6% | 6.3%^b^ | 12.4%^b^ | 11.7%^b^ | 9.4%^b^ | 34.9%^a^ |
| 14. Information is provided to staff, residents and representatives in a timely manner | 10.8% | 11.5% | 12.4% | 10.6% | 12.0% | 10.6% | 10.1%^a,b^ | 6.6%^a^ | 20.8%^b^ | 15.1%^a,b^ | 9.3%^a,b^ |
| 15. Issue reporting process is in place for continuous improvement, regulatory compliance and other relevant aspects of service | 4.0% | 8.0% | 4.3% | 6.9% | 7.2% | 5.3% | 1.9%^b^ | 5.1%^a,b^ | 14.3%^a^ | 9.4%^a,b^ | 5.8%^a,b^ |

ACSAA: Aged Care Standards and Accreditation Agency (operational until end of 2013). All the ACSAA reports published between 2011 and 2013 were included for analysis.

AACQA: Australian Aged Care Quality Agency (commenced operation in 2014). A stratified sample of AACQA reports published between 2014 and 2018 was included for analysis.

Different superscript letters denote significant difference in the percentage of reports mentioning the accreditation indicator between the ACSAA and AACQA reports, between RAC homes using EHR or paper-based information system, of small or large size and located in different states.

NSW: New South Wales, VIC: Victoria, QLD: Queensland, WA: Western Australia, SA: South Australia, TAS: Tasmania, NT: Northern Territory and ACT: Australian Capital Territory

EHR: electronic health records

# Appendix 2. A comparison of the percentage presence of a measurement criterion for the accreditation outcome 2.4 Clinical Care.

| **Accreditation indicator** | **Type of reports** | | **Type of information system** | | **Size of RAC homes** | | **State** | | | | |
| --- | --- | --- | --- | --- | --- | --- | --- | --- | --- | --- | --- |
|  | **ACSAA** | **AACQA** | **EHR** | **Paper** | **Small** | **Large** | **NSW** | **VIC** | **QLD** | **SA** | **ACT, NT, TAS, WA** |
| **Number of reports analysed** | **249** | **262** | **161** | **350** | **209** | **302** | **158** | **137** | **77** | **53** | **86** |
| 1. Residents and representatives are satisfied with the clinical care provided | 73.1%^b^ | 83.2%^a^ | 77% | 79% | 78% | 78% | 77% | 78% | 90% | 74% | 74% |
| 2. Management monitors clinical care through audits, clinical data analysis, monthly care plan reviews and consultation with the resident and/or their representative | 70.3%^b^ | 79.0%^a^ | 79% | 73% | 76% | 74% | 75% | 70% | 78% | 96% | 66% |
| 3. Residents’ care needs are assessed on entry to home | 60% | 66% | 58% | 65% | 65% | 62% | 52.5%^b^ | 70.8%^a,c^ | 76.6%^c^ | 66%^a,b,c^ | 54.7%^a,b^ |
| 4. Clinical care is provided according to residents' needs and preferences | 24.1%^b^ | 59.5%^a^ | 48% | 39% | 44% | 41% | 37% | 44% | 52% | 55% | 34% |
| 5. Processes (or system) exist to ensure that residents receive appropriate clinical care | 40.2%^b^ | 55.0%^a^ | 48% | 47% | 46% | 49% | 57% | 42% | 42% | 40% | 50% |
| 6. Comprehensive assessment, plan and evaluation (review) of clinical care is conducted | 65.1%^b^ | 54.2%^a^ | 58% | 60% | 62% | 58% | 56% | 63% | 57% | 57% | 65% |
| 7. Residents’ care needs are assessed on an ongoing basis | 26.1%^b^ | 49.2%^a^ | 37% | 39% | 37% | 38% | 41% | 39% | 44% | 36% | 27% |
| 8. In-depth care plans are developed and reviewed regularly | 51% | 47% | 49% | 49% | 50% | 49% | 50.6%^b, c^ | 36.5%^b, d^ | 59.7%^a, c^ | 28.3%^d^ | 69.8%^a^ |
| 9. Information relating to residents' health status is communicated via a variety of means such as handover, progress notes, email, communication diaries and health professional folders. | 37.3%^b^ | 46.6%^a^ | 43% | 42% | 42% | 42% | 43.0%^a, b^ | 45.3%^a, b^ | 55.8%^b^ | 32.1%^a, b^ | 29.1%^a^ |
| 10. Clinical incidents are reported, actioned appropriately and analysed to identify risks, trends, opportunities for improvement and the need for staff education | 12.0%^b^ | 43.9%^a^ | 32% | 27% | 26% | 30% | 22% | 32% | 38% | 21% | 31% |
| 11. Referral to medical and/or allied health professionals or transfer to hospital occurs in accordance with residents' needs and preferences and as required | 8.4%^b^ | 29.4%^a^ | 21% | 18% | 22% | 17% | 19.0%^a, b^ | 18.2%^a, b^ | 32.5%^b^ | 17.0%^a, b^ | 10.5%^a^ |
| 12. Changes in care are communicated with staff and other health professionals in a timely manner and care plan adjustments are made as required | 7% | 23% | 14% | 16% | 15% | 15% | 8.9%^b^ | 16.8%^a, b^ | 27.3%^a^ | 13.2%^a, b^ | 15.1%^a, b^ |
| 13. Staff have access to current information | 4% | 22% | 13% | 13% | 12% | 14% | 6.3%^b^ | 17.5%^a^ | 20.8%^a^ | 20.8%^a^ | 8.1%^a, b^ |
| 14. Staff are knowledgeable about individual resident care needs and preferences | 32.1%^b^ | 20.6%^a^ | 24% | 27% | 28% | 25% | 32.9%^b, c^ | 22.6%^a, b^ | 42.9%^c^ | 13.2%^a, b^ | 12.8%^a^ |
| 15. Before the completion of the care plan, an interim care plan derived from initial assessment is used | 14% | 19% | 19% | 16% | 16% | 18% | 9% | 15% | 38% | 25% | 12% |
| 16. The management team provide training and education to ensure staff are competent in the delivery of care recipients' care | 21% | 16% | 19% | 19% | 21% | 17% | 22% | 17% | 14% | 13% | 24% |
| 17. The home has appropriate supplies of equipment and resources that are maintained in good working order to meet the ongoing needs of care recipients | 3% | 6% | 4% | 5% | 4% | 5% | 8% | 3% | 1% | 2% | 5% |

ACSAA: Aged Care Standards and Accreditation Agency (operational until end of 2013). All the ACSAA reports published between 2011 and 2013 were included for analysis.

AACQA: Australian Aged Care Quality Agency (commenced operation in 2014). A stratified sample of AACQA reports published between 2014 and 2018 was included for analysis.

Different superscript letters denote significant difference in the percentage of reports mentioning the accreditation statements between the two types of reports, between RAC homes using EHR or paper-based information system, of small or large size and located in different states.

NSW: New South Wales, VIC: Victoria, QLD: Queensland, WA: Western Australia, SA: South Australia, TAS: Tasmania, NT: Northern Territory and ACT: Australian Capital Territory

EHR: electronic health records

# Appendix 3. The number and percentage of reports recorded failure in each of the 44 expected accreditation outcomes.

| **Expected accreditation outcome** | **ACSAA reports 2011-2013**  **(N=2,684)** | **AACQA reports 2014-2018**  **(N=2,876)** | **Total**  **(N=5,560)** |
| --- | --- | --- | --- |
| Overall failure rate of RAC homes* | 1.2% (33) | 4.6% (133) | 3.0% (166) |
| 1.1 Continuous improvement* | 0.1% (4) | 0.5% (14) | 0.3% (18) |
| 1.2 Regulatory compliance* | 0.04% (1) | 0.4% (12) | 0.2% (13) |
| 1.3 Education and staff development | 0.1% (4) | 0.3% (10) | 0.3% (14) |
| 1.4 Comments and complaints* | 0.04% (1) | 0.8% (22) | 0.4% (23) |
| 1.5 Planning and leadership | 0 | 0 | 0 |
| 1.6 Human resource management* | 0.4% (11) | 2.2% (64) | 1.3% (75) |
| 1.7 Inventory and equipment | 0.04% (1) | 0.1% (4) | 0.1% (5) |
| 1.8 Information systems* | 0.7% (19) | 1.8% (51) | 1.3% (70) |
| 1.9 External services | 0.04% (1) | 0.1% (3) | 0.1% (4) |
| 2.1 Continuous improvement* | 0.4% (10) | 1.0% (28) | 0.7% (38) |
| 2.2 Regulatory compliance | 0.1% (2) | 0.2% (6) | 0.1% (8) |
| 2.3 Education and staff development* | 0.3% (7) | 0.8% (23) | 0.5% (30) |
| 2.4 Clinical care* | 0.6% (17) | 1.9% (56) | 1.3% (73) |
| 2.5 Specialised nursing care needs* | 0.2% (6) | 0.6% (18) | 0.4% (24) |
| 2.6 Other health and related services | 0.1% (4) | 0.2% (7) | 0.2% (11) |
| 2.7 Medication management* | 0.5% (14) | 1.5% (42) | 1.0% (56) |
| 2.8 Pain management* | 0.3% (9) | 0.9% (27) | 0.6% (36) |
| 2.9 Palliative care* | 0 | 0.2% (7) | 0.1% (7) |
| 2.10 Nutrition and hydration | 0.3% (9) | 0.5% (14) | 0.4% (23) |
| 2.11 Skin care* | 0.2% (5) | 0.9% (26) | 0.6% (31) |
| 2.12 Continence management* | 0.2% (5) | 0.5% (15) | 0.4% (20) |
| 2.13 Behavioural management* | 0.3% (8) | 1.1% (33) | 0.7% (41) |
| 2.14 Mobility, dexterity and rehabilitation* | 0.04% (1) | 0.4% (11) | 0.2% (12) |
| 2.15 Oral and dental care | 0 | 0.03% (1) | 0.02% (1) |
| 2.16 Sensory loss | 0.04% (1) | 0.03% (1) | 0.04% (2) |
| 2.17 Sleep | 0 | 0.1% (4) | 0.1% (4) |
| 3.1 Continuous improvement* | 0.04% (1) | 0.3% (9) | 0.2% (10) |
| 3.2 Regulatory compliance* | 0.1% (4) | 0.9% (25) | 0.5% (29) |
| 3.3 Education and staff development | 0.1% (3) | 0.3% (9) | 0.2% (12) |
| 3.4 Emotional support* | 0 | 0.5% (13) | 0.2% (13) |
| 3.5 Independence | 0.04% (1) | 0.1% (3) | 0.1% (4) |
| 3.6 Privacy and dignity* | 0.3% (7) | 1.1% (32) | 0.7% (39) |
| 3.7 Leisure interests and activities* | 0.1% (3) | 0.6% (17) | 0.4% (20) |
| 3.8 Cultural and spiritual life | 0 | 0.1% (2) | 0.0% (2) |
| 3.9 Choice and decision-making | 0.1% (2) | 0.3% (8) | 0.2% (10) |
| 3.10 Resident security of tenure and responsibilities | 0 | 0.1% (3) | 0.1% (3) |
| 4.1 Continuous improvement* | 0.1% (2) | 0.5% (14) | 0.3% (16) |
| 4.2 Regulatory compliance | 0.1% (2) | 0.2% (7) | 0.2% (9) |
| 4.3 Education and staff development | 0.1% (3) | 0.2% (7) | 0.2% (10) |
| 4.4 Living environment* | 0.2% (6) | 1.1% (33) | 0.7% (39) |
| 4.5 Occupational health and safety* | 0.04% (1) | 0.3% (10) | 0.2% (11) |
| 4.6 Fire, security and other emergencies* | 0.04% (1) | 0.3% (10) | 0.2% (11) |
| 4.7 Infection control* | 0.04% (1) | 0.4% (12) | 0.2% (13) |
| 4.8 Catering, cleaning and laundry services* | 0.1% (4) | 0.9% (27) | 0.6% (31) |

ACSAA: Aged Care Standards and Accreditation Agency (operational until end of 2013). All the ACSAA reports published between 2011 and 2013 were included for analysis.

AACQA: Australian Aged Care Quality Agency (commenced operation in 2014). A stratified sample of AACQA reports published between 2014 and 2018 was included for analysis.

*denotes significant difference in the percentage of reports recorded failure between ACSAA and AACQA reports.

# Appendix 4. The ten RAC homes that failed more than once.

| **RAC home ID** | **State** | **Year of failure** | **Size of the RAC home** | **Type of system** | **No. of failure over years** | **No. of failed outcomes in the recent failure** | **Failed outcomes** | **Previous year of failure** | **Previous no. of failed outcomes** | **Previously failed outcomes** |
| --- | --- | --- | --- | --- | --- | --- | --- | --- | --- | --- |
| A | NT | 2013 | Large | EHR | 2 | 1 | 2.4 Clinical care | 2012 | 1 | 2.4 Clinical care |
| B | ACT | 2015 | Large | EHR | 2 | 2 | 1.8 Information systems  2.4 Clinical care | 2012 | 4 | 1.6 Human resource management  1.8 Information systems  2.5 Specialised nursing care needs  2.7 Medication management |
| C | QLD | 2016 | Large | Paper | 2 | 1 | 2.11 Skin care | 2015 | 7 | 1.6 Human resource management  1.8 Information systems  2.13 Behavioural management  3.6 Privacy and dignity  4.4 Living environment  4.7 Infection control  4.8 Catering, cleaning and laundry services |
| D | NSW | 2017 | Small | Paper | 2 | 2 | 1.2 Regulatory compliance  1.8 Information systems | 2015 | 5 | 1.2 Regulatory compliance  1.8 Information systems  2.7 Medication management  3.2 Regulatory compliance,  3.1 Resident security of tenure and responsibilities |
| E | QLD | 2017 | Small | EHR | 2 | 15 | 1.6 Human resource management  1.8 Information systems  2.1 Continuous improvement  2.3 Education and staff development  2.4 Clinical care  2.6 Other health and related services  2.7 Medication management  2.8 Pain management  2.10 Nutrition and hydration  2.11 Skin care  2.12 Continence management  2.13 Behavioural management  3.4 Emotional support  3.6 Privacy and dignity  4.4 Living environment | 2015 | 3 | 2.4 Clinical care  4.1 Continuous improvement  4.4 Living environment |
| F | WA | 2017 | Large | EHR | 3 | 1 | 2.4 Clinical care | 2017 | 6 | 1.6 Human resource management  1.8 Information systems  2.7 Medication management  2.8 Pain management  3.4 Emotional support  4.4 Living environment |
|  |  |  |  |  |  |  |  | 2016 | 13 | 1.1 Continuous improvement  1.4 Comments and complaints  1.6 Human resource management  1.8 Information systems  2.1 Continuous improvement  2.4 Clinical care  2.7 Medication management  2.8 Pain management  2.13 Behavioural management  3.4 Emotional support  3.6 Privacy and dignity  4.4 Living environment  4.5 Occupational health and safety |
| G | NSW | 2018 | Large | EHR | 2 | 5 | 1.6 Human resource management  1.8 Information systems  2.4 Clinical care  2.13 Behavioural management  3.2 Regulatory compliance | 2018 | 9 | 1.6 Human resource management  1.8 Information systems  2.4 Clinical care  2.5 Specialised nursing care needs  2.7 Medication management  2.8 Pain management  2.13 Behavioural management  4.2 Regulatory compliance  4.7 Infection control |
| H | NSW | 2018 | Large | Paper | 2 | 3 | 1.4 Comments and complaints  1.6 Human resource management  2.5 Specialised nursing care needs | 2017 | 9 | 1.4 Comments and complaints  1.6 Human resource management  1.8 Information systems  2.7 Medication management  2.10 Nutrition and hydration  2.13 Behavioural management  3.2 Regulatory compliance  3.6 Privacy and dignity  4.4 Living environment |
| I | NSW | 2018 | Large | Paper | 2 | 1 | 3.2 Regulatory compliance | 2018 | 14 | 1.6 Human resource management  1.8 Information systems  2.1 Continuous improvement  2.2 Regulatory compliance  2.3 Education and staff development  2.4 Clinical care  2.5 Specialised nursing care needs  2.7 Medication management  2.8 Pain management  2.1 Nutrition and hydration  2.11 Skin care  2.13 Behavioural management  2.14 Mobility, dexterity and rehabilitation  3.2 Regulatory compliance |
| J | QLD | 2018 | Small | EHR | 2 | 6 | 2.4 Clinical care  2.6 Other health and related services  2.11 Skin care  4.2 Regulatory compliance  4.4 Living environment  4.6 Fire, security and other emergencies | 2018 | 6 | 2.4 Clinical care  2.6 Other health and related services  2.11 Skin care  4.2 Regulatory compliance  4.4 Living environment  4.6 Fire, security and other emergencies |
